# Inpatient palliative chemotherapy is associated with high mortality and aggressive end-of-life care in patients with advanced solid tumors and poor performance status

**DOI:** 10.1186/s12904-019-0427-4

**Published:** 2019-05-20

**Authors:** Vitor Fiorin de Vasconcellos, Renata RCC Bonadio, Guilherme Avanço, Marcelo Vailati Negrão, Rachel Pimenta Riechelmann

**Affiliations:** 1 Medical Oncology Department, Instituto do Cancer do Estado de São Paulo (ICESP), Avenida Dr. Arnaldo, 251, Cerqueira César, São Paulo, 01246-000 Brazil; 2 Department of Thoracic/Head and Neck Medical Oncology, The University of Texas, MD Anderson Cancer Center, 1515 Holcombe Blvd, Unit 432, Houston, TX 77030 USA; 3 Department of Clinical Oncology, AC Camargo Cancer Center, R. Prof. Antônio Prudente, 211 – Liberdade, São Paulo, SP 01509–010 Brazil

**Keywords:** Palliative, Chemotherapy, Metastasis, Cancer, Hospitalization, Medical futility, Prognosis, Retrospective study, Survival rate, Latin America

## Abstract

**Background:**

The benefit of palliative chemotherapy (PC) in patients with advanced solid tumors and poor performance status (ECOG-PS) has not been prospectively validated, which makes treatment decision challenging. We aimed to evaluate the overall survival, factors associated with early mortality, and adoption of additional procedures in hospitalized patients with advanced cancer and poor ECOG-PS treated with PC.

**Methods:**

We analyzed a retrospective cohort of patients with advanced cancer treated with PC during hospitalization at an academic cancer center in Brazil from 2014 to 2016. Eligibility criteria included: ECOG-PS 3–4 and start of first-line PC; or ECOG-PS ≥ 2 and start of second or subsequent lines. Primary endpoint was 30-day survival from start of PC. Kaplan-Meier method was used for survival estimates and Cox regression for factors associated with 30-day mortality.

**Results:**

Two hundred twenty-eight patients were eligible. 21.9, 66.7 and 11.4% of patients had ECOG-PS 2, 3 and 4, respectively. 49.6% had gastrointestinal tumors. Median follow-up was 49 days (range 1–507). 98.2% of patients had died, 32% during the index hospitalization. The 30-day and 60-day survival rates were 55.7 and 38.5%, respectively. 30% of patients were admitted to the intensive care unit. In a multivariable analysis, ECOG-PS 3/4 (HR 2.01; *P* = 0.016), hypercalcemia (HR 2.19; *P* = 0.005), and elevated bilirubin (HR 3.17; *P* <  0.001) were significantly associated with 30-day mortality.

**Conclusions:**

Patients with advanced cancer and poor ECOG-PS had short survival after treatment with inpatient PC. Inpatient PC was associated with aggressive end-of-life care. Prognostic markers such as ECOG-PS, hypercalcemia and elevated bilirubin can contribute to the decision-making process for these patients.

## Background

Palliative chemotherapy (PC) is a non-curative treatment given to cancer patients diagnosed with unresectable or metastatic disease to improve patients’ symptomatic control and/or quality of life, or to prolong survival [1, 2]. However, these potential benefits of treatment must be weighed against the risks for potentially serious and life-threatening adverse events such as neutropenic fever, major bleeding, renal failure, colitis, hepatic dysfunction, among others [3].

Due to the lack of robust predictive biomarkers of benefit from conventional cytotoxic chemotherapy, general prognostic markers such as performance status and patient comorbidities are usually used to select patients that are eligible for this type of therapy [4]. One of the well-established methods to assess cancer patients’ performance status (PS) is the Eastern Cooperative Oncology Group Performance Status (ECOG-PS), an ordinal 5-points scale with increasing scores indicating more severe disability [1].

The American Cancer Society of Clinical Oncology (ASCO) previously published recommendations against the use of cancer-directed therapy for patients with solid tumors and poor ECOG-PS (i.e., ECOG-PS ≥ 3 defined as “capable of only limited self-care, confined to bed or chair more than 50% of walking hours”) [5]. This was based on the limited inclusion of these patients in clinical trials as well as observational studies correlating poor ECOG-PS to lower response rate, poor treatment tolerance and shorter overall survival [5–7]. Additionally, this guideline considers a 30-day mortality rate of 20–50% to be excessive.

On the other hand, a previous retrospective analysis of 240 consecutive patients conducted in our institution showed that 48.6% of patients with metastatic colorectal cancer with an ECOG-PS 3–4 achieved some clinical benefit in terms of symptom improvement, experienced a low rate of serious adverse events (21.7%) and overall survival was longer compared to patients treated with best supportive care (BSC) (OS 6.8 versus 2.3 months, respectively) [8].

Another retrospective analysis of 199 patients with advanced cancer who started PC while hospitalized (breast cancer: 23%; small cell lung cancer: 22%; non-small cell lung cancer: 16%), showed a hospital discharge rate of 77% and 72% of patients were able to receive subsequent cycles of chemotherapy [9].

Therefore, a better understanding of the consequences of the use of PC in those patients is required to avoid unnecessary or potentially harmful therapies and misguided decisions in end-of-life care [10–13]. Identifying the factors associated with a higher risk of early mortality would contribute to substantiate decisions regarding active treatment or best support of care.

In this study, we aimed to evaluate the outcomes of patients with advanced cancer and poor ECOG-PS treated with inpatient PC, including the overall survival, factors associated with early mortality, and the rate of invasive procedures performed. We hypothesized that inpatient PC in this scenario would be associated with short overall survival, indirectly compared with ECOG-PS 0 or 1 cancer patients, and aggressive medical care near the end of life.

## Methods

### Patient accrual and data collection

This was a retrospective study of consecutive hospitalized patients with advanced and incurable solid tumors and poor ECOG-PS who were treated with PC during hospitalization at a large academic and public cancer center (*Instituto do Câncer do Estado de São Paulo* - ICESP), between January 2014 and September 2016.

Advanced and incurable solid tumors were defined as recurrent or metastatic tumors for which no treatment with curative intent was available. Therapies with curative intent include complete surgical resection, definitive chemoradiation, or systemic chemotherapy for germ cell tumors. We defined poor performance status as ECOG-PS ≥2 on the first day of treatment with PC. ECOG-PS was available for all patients in the institution per electronic patient chart. The cancer center where the study took place, ICESP, is one of the largest cancer centers of Latin America, with approximately 10,000 new cancer patients per year and 499 beds for patients’ hospitalization.

Patients were included if they had histologically confirmed advanced solid tumors, and had ECOG-PS 3–4 at the time of starting frontline PC, or had ECOG-PS ≥ 2 at the time of starting second or later lines of chemotherapy. The rationality for these inclusion criteria was to represent poor ECOG-PS patients that are usually not included in clinical trials. Patients with ECOG-PS 2 receiving first-line chemotherapy were not included since this is often considered a standard of care. Solid tumors were considered all non-hematological malignancies.

Exclusion criteria included highly chemo-sensitive histologies (germ cell tumors, ovarian serous adenocarcinoma, and small cell lung cancer) and primary tumors of the central nervous system (CNS). Patients with primary CNS tumors were excluded because they frequently present poor ECOG-PS due to neurologic deficits even at disease diagnosis. To obtain our sample, we reviewed data from all consecutive patients that received chemotherapy during hospitalization according to the hospital records and included those that met eligibility criteria. The medical records were available at the cancer center (ICESP) database. Accessing the database required a permission, which was obtained with the Research Center of ICESP.

Data was collected from the electronic medical records and included: age, gender, primary tumor site, clinical stage (based on imaging studies), number of metastatic sites, main symptom(s) at hospitalization, line of chemotherapy and regimen (single agent versus combination), Charlson Comorbidity Index score, and body mass index. Charlson Comorbidity Index is a scale used to predict mortality based on patient comorbidities [14]. Main symptom(s) at hospitalization were the symptoms related to the reason of hospitalization (e.g.: abdominal pain and obstipation in case of malignant bowel obstruction). Nausea, vomiting, diarrhea, obstipation and abdominal pain were grouped as gastrointestinal symptoms. Neurologic symptoms included headache, seizure, focal neurologic deficits, and altered level of consciousness.

Laboratory tests at admission were reviewed to determine the hemoglobin (Hb) levels, total leukocyte and lymphocyte count, creatinine (Cr), alanine aminotransferase (ALT), aspartate aminotransferase (AST), total bilirubin (Bb), and calcium levels. Data on additional procedures used during hospitalization and place of death were also collected. Additional procedures recorded were blood transfusions, palliative radiotherapy, surgical procedures and/or admission to the intensive care unit (ICU).

The primary endpoint of the study was 30-day survival rate from the start of the first inpatient chemotherapy cycle. Secondary endpoints included median survival, 60-day survival rate from the start of the first inpatient chemotherapy cycle, prognostic factors associated with survival, rate of additional procedures performed, and hospital discharge rate after starting chemotherapy.

The prognostic factors evaluated were sex, age, primary tumor site, number of sites of metastases (less than or equal to 2 vs greater than 2), main symptom that led to hospitalization, ECOG-PS (3–4 vs 2), body mass index and Charlson index (≤ 6 vs > 6 points), and laboratory abnormalities. The cutoff value for the number of sites of metastases was chosen based on previous studies suggesting that the presence of more than 2 metastatic sites is associated with shorter overall survival [15, 16]. As for the Charlson index, we decided to compare ≤6 vs > 6 points because most patients would have a score of at least 6 due to the diagnosis of a metastatic solid tumor.

The laboratory tests were categorized according to the presence or absence of the following laboratory abnormalities: anemia (defined by Hb < 10 g/dL), leukocytosis (leukocytes ≥10.000 per mm^3^), lymphopenia (lymphocytes ≤1500 per mm^3^), hypercalcemia (defined by ionized calcium ≥5.3 mEq/L or total corrected calcium ≥10.2 mg/dL), elevated liver function tests (LFT) (defined by AST or ALT ≥2.5 times upper limit of normal), renal impairment (defined by Cr ≥ 1.5 mg/dL), and elevated bilirubin (defined by total Bb ≥ 1.5 mg/dL). Both clinical and laboratory variables were categorized based on clinical relevance for interpretation of the results in the clinical setting. Information on the clinical variables was available for all patients since it is systematically registered in electronic records during hospitalization at ICESP. For the laboratory tests, the median value was used for missing value imputation. The rates of missing data for the laboratory variables ranged from 0 to 17%.

### Statistical analysis

Descriptive statistics were used to summarize patient characteristics. The absolute and relative frequencies of clinical and demographic data were tabulated. Qualitative variables were presented as proportions and quantitative variables were presented as median and respective ranges.

Survival analysis was performed using the Kaplan-Meier method. Factors associated with 30-day mortality were evaluated with univariable and multivariable analysis by using Cox regression. Prognostic factors with *P* ≤ 0.10 in univariable analysis and with no association between each other were included in the multivariable model. The association between categorical variables was evaluated by using χ2 test. A two-tailed *P* value ≤0.05 was considered statistically significant. Statistical analyses were performed using Stata software, version 14 (StataCorp, Texas, USA).

## Results

### Patients’ characteristics

**Fig. 1:**
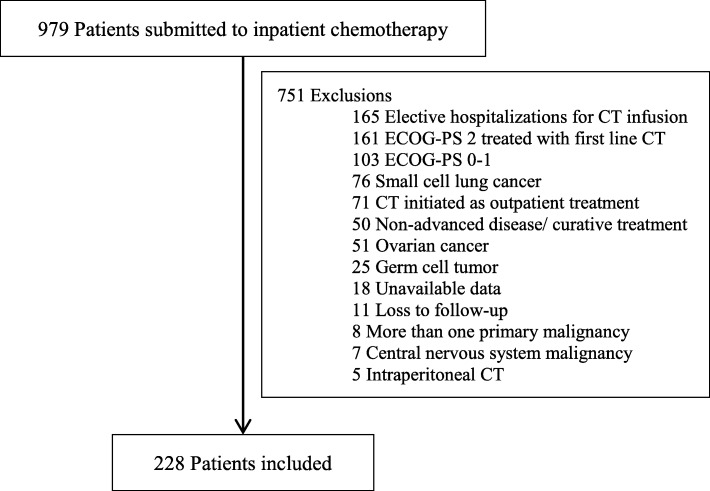
CONSORT diagram

A total of 979 patients with solid tumors received PC during hospitalization between 2014 and 2016. Of these, 228 consecutive patients fulfilled the eligibility criteria and were included in the analysis. The CONSORT diagram is presented in Fig. 1.

Median age was 56 years (range 21–79). The majority of patients were female (58%) and chemotherapy-naïve (66%). The most common primary tumor sites were gastrointestinal (49.6%) and breast (18.4%). The proportions of ECOG-PS 2, 3 and 4 were 21.9, 66.7, and 11.4%, respectively. The majority of patients had metastatic disease at presentation (*N* = 223; 97.8%), and only 5 patients (2.2%) had a locally advanced disease not amenable for treatment with curative intent. Patients were predominantly treated with a combination chemotherapy regimen (*N* = 173; 76%).

Seventy-one patients (31.1%) were hospitalized because of gastrointestinal symptoms, including 40 patients (17.5%) with malignant bowel obstruction (*N* = 40; 17.5%). Other common causes of admission included dyspnea, pain, and sepsis.

Patient characteristics are summarized in Table 1.

**Table 1:** Patient characteristics

	N	%
Male gender	95	41.7
Age: median (range)	56 (21–79)	
Primary tumor site		
Gastrointestinal	113	49.6
Colorectal	45	19.7
Gastric	38	16.6
Pancreatic/Biliary	13	5.7
Others	17	7.4
Breast	42	18.4
NSCLC	25	11
Sarcoma	13	5.7
Gynecologic	11	4.8
Genitourinary	9	3.9
Head and Neck	7	3.1
Skin (squamous cell carcinoma)	5	2.2
Unknown primary	3	1.3
Number of metastatic sites		
≤ 2	96	42.1
> 2	132	57.9
Main symptom at hospitalization		
Pain	35	15.4
Dyspnea	51	22.4
Infection-related symptoms	26	11.4
Bleeding/ Symptomatic anemia	15	6.6
Gastrointestinal symptoms	71	31.1
Neurologic symptoms	11	4.8
Fatigue	19	8.3
Chemotherapy line		
First line	152	66.6
Second line or greater	76	33.3
Chemotherapy regimen		
Single agent	55	24.1
Combination regimen	173	75.8
ECOG-PS		
2	50	21.9
3	152	66.7
4	26	11.4
Anemia^1^	119	52.2
Leukocytosis^2^	85	37.3
Lymphopenia^3^	86	37.7
Hypercalcemia^4^	23	10.1
Renal impairment^5^	21	9.2
Elevated liver function tests^6^	43	18.9
Total bilirubin elevation^7^	33	14.5
BMI		
Underweight^8^	32	14
Normal^9^	123	53.9
Overweight^10^	37	16.2
Obesity^11^	36	15.8
Charlson Index > 6	63	27.6

Abbreviations: *NSCLC* non-small cell lung cancer, *ECOG-PS* Eastern Cooperative Oncology Group Performance Status, *BMI* body mass index

^**1**^Hemoglobin < 10 g/dL; ^**2**^White Blood Cell count ≥10.000/mm^3^; ^**3**^Lymphocytes count ≤1500/mm^3^; ^**4**^Ionized Calcium ≥5.3 mEq/L or Total Corrected Calcium ≥10.2 mg/dL; ^**5**^Creatinine ≥ 1.5 mg/dL**;** ^6^Aspartate Aminotransferase or Alanine Aminotransferase ≥2.5 times upper limit of normal; ^**7**^Total Bilirubin ≥1.5 mg/dL; ^**8**^BMI < 18.5; ^**9**^BMI 18.5 to 24.9; ^**10**^BMI 25 to 29.9; ^**11**^BMI ≥ 30

### Outcomes

Median follow–up was 49 days (range 1–507 days). Two hundred and twenty-four patients had died (98.2%), and median overall survival was 38.5 days. 30-day and 60-day survival rates were 55.7 and 38.5%, respectively. Seventy-three patients (32%) died during the same admission in which PC was started. Thirty-one patients (13.8%) died in the intensive care unit (ICU), twenty-seven patients (12%) died in an inpatient hospice facility and two patients (0.8%) died at home.

Median duration of index hospitalization was 16 days (range 2–87 days). Eighty-six patients (37%) required invasive procedures during the index hospitalization, which included 68 patients (30%) that required intensive care unit admission and 37 (16%) that underwent surgical procedures. One hundred and three patients (45%) received blood transfusions, and 22 patients (9.6%) received palliative radiotherapy. Approximately 68% of patients (*N* = 155) were discharged from the hospital after receiving PC, and 100 patients (43.8%) received at least one more cycle of PC as an outpatient after the index hospitalization. Outcomes of interest are summarized in Table 2.

**Table 2:** Summary of the outcomes of interest

Outcome	*N* = 228
Deaths – N (%)	224 (98.2)
30-day survival rate - %	55.7
60-day survival rate - %	38.5
Median overall survival - days	38.5
Death during the index hospitalization – N (%)	73 (32)
Place of death – N (%)	
Intensive care unit (ICU)	31 (13.8)
Hospital (except ICU)	164 (73.2)
Inpatient hospice facility	27 (12)
Home	2 (0.8)
Additional procedures – N (%)	
ICU admission	68 (30)
Surgical procedures	37 (16)
Blood transfusion	103 (45)
Palliative radiotherapy	22 (9.6)

Abbreviations: *ICU* intensive care unit

### Factors associated with 30-day mortality

In univariable analysis, 30-day mortality was positively correlated with presence of gastrointestinal symptoms at admission (harzard ratio [HR]: 2.04, 95% confidence interval [CI] 1.07–3.88, p 0.029), ECOG-PS (3–4 vs 2) (HR 2.05, 95% CI 1.17–3.62, *P* = 0.012), hypercalcemia (HR 2.09, 95% CI 1.20–3.63, *P* = 0.008), elevated LFTs (HR 1.84, 95% CI 1.17–2.90, *P* = 0.008) and elevated bilirubin (HR 3.07, 95% CI 1.94–4.84, *P* = < 0.001).

Among these variables, only ECOG-PS, hypercalcemia, and elevated bilirubin met inclusion criteria for the multivariable model. Gastrointestinal symptoms and elevated LFTs were not included because they were positively correlated with elevated bilirubin (χ2 *P* = 0.003; and χ2 *P* <  0.001, respectively). In the multivariable analysis, three prognostic factors were independently associated with 30-day mortality: ECOG-PS (3–4 vs 2) (HR 2.01, 95% CI 1.14–3.53, *P* = 0.016), hypercalcemia (HR 2.19, 95% CI 1.26–3.80, *P* = 0.041), and elevated bilirubin (HR 3.17, 95% CI 2.00–5.01, *P* < 0.001).

The results for the univariable and multivariable analyses are summarized in Tables 3 and 4, respectively.

**Table 3:** Univariable analyses of factors associated with 30-day mortality

	HR (95% CI)	*P* value
Female gender	1.25 (0.83–1.87)	0.270
Age	1.00 (0.98–1.01)	0.898
Primary tumor site		
Cutaneous squamous-cell carcinoma	(reference)	
Gastrointestinal	1.32 (0.32–5.44)	0.693
Breast	1.15 (0.26–5.01)	0.845
Head and Neck	2.75 (0.53–14.23)	0.226
Genitourinary	0.52 (0.07–3.69)	0.515
Gynecologic	1.00 (0.18–5.48)	0.996
Lung	0.92 (0.19–4.25)	0.915
Sarcoma	0.87 (0.16–4.49)	0.871
Unknown primary	0.90 (0.08–9.94)	0.933
Number of metastatic sites (> 2 vs ≤ 2)	0.91 (0.66–1.35)	0.663
Main symptom at hospitalization		
Pain	(reference)	
Dyspnea	1.04 (0.50–2.17)	0.896
Infection-related symptoms	1.14 (0.49–2.64)	0.753
Bleeding/ Symptomatic anemia	0.53 (0.15–1.90)	0.336
Gastrointestinal symptoms	2.04 (1.07–3.88)	0.029
Neurologic symptoms	1.79 (0.67–4.78)	0.243
Fatigue	1.79 (0.77–4.16)	0.171
ECOG-PS (3–4 vs 2)	2.05 (1.17–3.62)	0.012
Anemia (yes vs no)	1.05 (0.71–1.55)	0.792
Leukocytosis (yes vs no)	1.20 (0.80–1.79)	0.364
Lymphopenia (yes vs no)	1.07 (0.72–1.60)	0.710
Hypercalcemia (yes vs no)	2.09 (1.20–3.63)	0.008
Renal impairment (yes vs no)	1.20 (0.62–2.31)	0.573
Liver enzymes elevation (yes vs no)	1.84 (1.17–2.90)	0.008
Total bilirubin elevation (yes vs no)	3.07 (1.94–4.84)	< 0.001
Body mass index		
Underweight	(reference)	
Normal	1.07 (0.58–1.97)	0.810
Overweight	1.09 (0.52–2.26)	0.817
Obesity	1.25 (0.61–2.59)	0.531
Charlson Index > 6	1.27 (0.83–1.94)	0.253

Abbreviations: *ECOG-PS* Eastern Cooperative Oncology Group Performance Status, *HR* hazard ratio, *CI* confidence interval

^**1**^Univariable Cox regression

**Table 4:** Multivariable analyses of factors associated with 30-day mortality

	Adjusted HR (95% CI)	*P* value
ECOG-PS (3–4 vs 2)	2.01 (1.14–3.53)	0.016
Hypercalcemia (yes vs no)	2.19 (1.26–3.80)	0.005
Total bilirubin elevation (yes vs no)	3.17 (2.00–5.01)	< 0.001

Abbreviations: *ECOG-PS* Eastern Cooperative Oncology Group Performance Status, *HR* hazard ratio, *CI* confidence interval

^**1**^Multivariable Cox regression. Adjusted variables: ECOG-PS, hypercalcemia, and elevated bilirubin

## Discussion

Our results showed that hospitalized patients with advanced cancers (with the exclusion of highly chemo-sensitive histologies and primary CNS tumors) and poor performance status had a low survival rate after receiving PC. Median OS was 38.5 days, and 30- and 60-day survival rates were 55.7 and 38.5%, respectively, highlighting that these patients had very poor outcomes regardless if they had initiated active cancer treatment or, in some cases, had received invasive life support interventions (e.g., ICU admission and surgical procedures).

Previous phase II/III clinical trials and meta-analysis have shown that cytotoxic chemotherapy improved survival and quality of life compared to BSC alone across different primary solid tumors [17–19]. However, these results cannot be extrapolated to indicate inpatient PC to poor performance status patients because this population is often excluded or underrepresented in clinical trials [20].

A Spanish single-center study with eligibility criteria similar to ours analyzed 92 patients and showed that mOS was 33 days from the last course of chemotherapy [21]. A Canadian single-center cohort with 199 patients reported mOS of 4.5 months from the date of starting chemotherapy in the inpatient setting [22]. One possible explanation for the heterogeneous findings in Canada is that 22% of their patients had small-cell lung cancer, a recognized highly chemo-sensitive histology that was excluded from our analysis. Our results were consistent with the majority of literature that shows a short survival for poor performance status patients after receiving inpatient PC.

Importantly, the use of chemotherapy near the end of life has been associated with more invasive procedures near death, including ICU admissions, delays in hospice referrals, and increased treatment costs [23–25]. In a prospective cohort study evaluating 386 terminally ill cancer patients, 11% of the patients that received PC died in the ICU in comparison with 2% of those who did not receive PC (*P* = 0.02) [25]. In our cohort, 37% of patients underwent invasive procedures, 30% underwent ICU admission, and 13.8% died in the ICU. These findings raised concern for futility and potential harm in end-of-life care (e.g. death in ICU) when adopting aggressive medical interventions in patients with advanced disease and poor ECOG-PS. In addition, limited resources available in the Brazilian public health system raise yet another reason to avoid not cost-effective and potentially futile interventions.

A combination of several factors has been described as a possible reason for why medical oncologists offer PC to poor performance status patients. These factors include: (i) physicians overestimate prognosis of metastatic cancer patients [26]; (ii) limited evidenced-based treatment recommendations in this scenario [27]; (iii) patients’ and families’ expectations to receive anti-cancer therapy [23]; (iv) young or middle-aged patients [28]; (v) absence of palliative care team participating in the patients care [29]; and (vi) care at an academic/ teaching hospital [24]. Our cohort was composed mainly of treatment naïve patients who were admitted in an academic cancer center due to symptoms related to advanced solid tumor disease. These characteristics could, at least in part, explain the high rates of inpatient PC and invasive procedures.

Our findings suggest an over-prescription of PC in this population because almost half of the patients (44.3%) died within 30 days of starting treatment. As mentioned previously, ASCO’s recommendation supports that 20–50% 30-day mortality rate after starting chemotherapy is excessive and requires revision of patient selection criteria for this type of treatment [6, 30, 31]. Moreover, ASCO lists the reduction of chemotherapy overuse in patients with poor ECOG-PS as one of the top five priorities to improve patient care and reduce health care costs [6].

Valuable efforts have been made in an attempt to determine prognostic markers that can help identify the patients with very short life expectancy, and therefore, not likely to benefit from PC [32]. In our study, hyperbilirubinemia was the strongest prognostic factor associated with 30-day mortality in the multivariable analysis. This finding is likely due to the correlation between increased bilirubin and liver failure, and because half of our patients had a primary gastrointestinal tumor, which commonly metastasizes to the liver. Also, ECOG-PS 3–4 and hypercalcemia were correlated with shorter survival.

Another retrospective study conducted at ICESP with predominantly outpatients reinforce that ECOG-PS 3–4 was a predictor of 90-mortality. In contrast to our results, Caires-Lima et al. cohort found that elevated creatinine was a statistically significant predictor factor of early mortality [33]. Validated prognostic tools were shown to predicted survival and can be useful in the evidence-based cancer treatment decision process [34]. Although this represents a promising approach, this strategy still requires prospective validation for general clinical use. More importantly, these prognostic markers still remain to be validated with more recent treatment approaches, such as tyrosine kinase inhibitors and immune checkpoint inhibitors, where treatment is usually much better tolerated and carries lower rates of grade 3–4 adverse events [35–37].

Our study has inherent limitations due to its retrospective nature. Other potential limitations are: (i) the lack of a control group; (ii) there was no systematic report of treatment related toxicities precluding the data collection; (iii) we were not able to obtain information related to quality of life and radiologic response; (iv) our study included a large variety of cancer types, with heterogeneous prognoses and a high proportion of stomach and pancreatic/biliary tumors, which could be associated with poor prognosis and may have contributed to a worse outcome in the population; and (v) unmeasured confounding factors (e.g. disease burden and high volume of metastatic disease) are also a potential source of bias.

On the other hand, the strengths of our cohort include: (i) its robust sample size, which, to the best of our knowledge, is the largest single-center cohort of patients with advanced cancer and poor performance status who started PC during hospitalization; (ii) our broad inclusion criteria allow a great representation of this population and a proper evaluation of risk factors associated with early mortality; (iii) we analyzed real-world data, which is potentially generalizable to other public academic institutions; (iv) the assistance for hospitalized patients with advanced malignancies may be influenced by local culture; and (v) we provide data on the oncologic care for this Latin America population, which is scarcely represented in previous studies.

Based on our results, we suggest that physicians should be aware of the outcomes of inpatient PC in this scenario and the factors associated with early mortality to allow better decision-making. Importantly, this information should also be shared with patients and their families to allow more informed decisions.

A multicenter and international exchange of experiences would be helpful in order to build a collaborative line of work [38], concentrating efforts to assess the real magnitude of the clinical benefit of PC and to increase the cost-effectiveness of active cancer treatments, especially in the limited resources scenario of developing countries [39].

## Conclusions

Our study showed that patients with advanced solid tumors and poor performance status treated with inpatient PC had a poor prognosis and short survival. PC in the inpatient setting is associated with aggressive end-of-life care, including ICU admission. Elevated bilirubin, hypercalcemia, and ECOG-PS 3–4 are associated with 30-day mortality and may be used to aid in treatment decisions for this patient population.

## Abbreviations

ALT: alanine aminotransferase
ASCO: American Society of Clinical Oncology
AST: aspartate aminotransferase
Bb: total bilirubin
BSC: best supportive of care
CNS: central nervous system
Cr: creatinine
ECOG-PS: Eastern cooperative oncology group performance status
Hb: hemoglobin
ICESP: *Instituto do Câncer do Estado de São Paulo*
ICU: intensive care unit
LFT: liver function tests
OR: Odds Ratio
PC: Palliative chemotherapy
